# Structural Characteristics and Hypolipidemic Activity of Theabrownins from Dark Tea Fermented by Single Species *Eurotium cristatum* PW-1

**DOI:** 10.3390/biom10020204

**Published:** 2020-01-30

**Authors:** Yue Xiao, Maoyun Li, Yanping Wu, Kai Zhong, Hong Gao

**Affiliations:** 1 College of Biomass Science and Engineering and Healthy Food Evaluation Research Center, Sichuan University, Chengdu 610065, China; scu_xyue@163.com (Y.X.); MYLeecheer@163.com (M.L.); wyp9202@163.com (Y.W.);; 2 Key Laboratory of Food Science and Technology of Ministry of Education of Sichuan Province, Sichuan University, Chengdu 610065, China

**Keywords:** theabrownins, fractionated, *E. cristatum* fermented loose tea, structural characteristics, hypolipidemic activity

## Abstract

Recently, studies on theabrownins (TBs), the main bioactive polymeric pigments found in dark tea, have received increasing attention for its health effects. Thus far, information on their structural characteristics is unclear. In the present study, theabrownins were isolated from single species *Eurotium cristatum* PW-1-fermented loose tea and their structural and hypolipidemic characteristics were studied for the first time. The theabrownins were fractionated by their molecular weights and were then analyzed. Ultraviolet–visible spectrophotometry (UV-Vis) and Flourier transformation infrared spectroscopy (FT-IR) showed that they were polymerized phenolic substances containing abundant hydroxy and carboxyl groups. All theabrownin samples exhibited hypolipidemic activity in high-fat zebrafish; among which TBs-10-30k sample, decreased lipid level in high-fat zebrafish to 51.57% at 1000 μg/mL, was most effective. It was found that TBs-10-30k was a type of amorphous and thermostable polymer with slice shape and smooth surface under scanning electron microscope (SEM). Atomic force microscope (AFM) analysis showed that it had island-like structure because of aggregation of theabrownin molecules. Pyrolysis-gas chromatography-mass spectrometry (Py-GC-MS) analysis further showed that the main pyrolytic products of TBs-10-30k were hexadecanoic acid (33.72%), phenol (14.90%), and eicosane (12.95%), indicating TBs-10-30k was mainly composed of phenols, lipids, saccharides, and proteins. These results not only facilitate subsequent identification of theabrownins, but also provide insights into the applications of theabrownins in functional foods.

## 1. Introduction

In addition to water, tea is one of the most popular beverages consumed worldwide because of its many health-promoting benefits, such as antioxidation, anticancer, anti-inflammatory, and hypolipidemic activities [1,2,3]. Tea contains a number of compounds, including the polyphenolic compounds, which are recognized as the main functional components in various types of teas. Catechins, which are the predominant polyphenolic compounds in green tea, can be oxidized during fermentation to produce different oxidized polyphenols, including theaflavins, thearubigins, and theabrownins [4]. Theaflavins and thearubigins are present in high concentrations in fully fermented black tea, whereas theabrownins are the main polyphenolic compounds in post-fermented dark teas, such as Fuzhuan brick tea and Pu-erh tea [4,5,6]. With the growing popularity of dark tea in China, the theabrownins, one of the main bioactive macromolecules in the dark tea, have received increasing attention because of its health benefits.

In the past few years, studies have provided important information on the functions and bioactivities of theabrownins, demonstrating that they can inhibit the activity of α-glucosidase in vitro, decrease blood glucose levels and increase glucose tolerance in mice under hyperglycemic stress [7]. Additionally, they exhibit anti-osteoporotic [8], anti-tumor [9,10,11], and hypolipidemic effects in vitro and in vivo [12,13,14,15]. Nonetheless, because of its complexity, the physicochemical properties of theabrownins are rarely studied. Since increasing attention has been paid to the health-promoting effects of post-fermented teas, theabrownins, which are the main bioactive compounds in these teas, deserve more investigations. Based on a few reported studies, theabrownins are a type of brown or reddish-brown pigment that are soluble in water but are insoluble in ethyl acetate, *n*-butyl alcohol or other organic solvents [12,13]. In addition, theabrownins are heterogeneous macromolecules that polymerized, condensed, coupled, and oxidized from catechins, theaflavins, and thearubigins with other compounds, such as proteins, lipids, polysaccharides, and some other components [4,16]. Because of their condensed nature, the molecules of theabrownins cannot be easily separated by chemical methods, the structural characteristics of theabrownins remain unclear, thus their applications remain limited.

It is observed that the molecular weights and the different sources of theabrownins have an impact on their structure and composition [16,17]. Therefore, researches on relatively pure and specific theabrownins are necessary. In our previous study, *Eurotium cristatum* fermented loose tea, a type of fungal fermented dark tea, has exhibited potent hypolipidemic activity in high-fat-induced zebrafish, which is related to the activity of theabrownins [18]. However, the physiochemical properties of theabrownins are unknown. Therefore, in the present study, theabrownins were isolated from the specific *E. cristatum* fermented loose tea and their structural characteristics were studied. 

Nowadays, obesity has progressively become a public health problem that needs to be addressed; effective ways that can prevent overweight and obesity are therefore needed, especially for the development of natural products with lipid-lowering activity. Herein, hypolipidemic activity of theabrownins and fractionated theabrownins with different molecular weights were evaluated using high-fat induced obesity zebrafish, and in-depth structural characteristics of the most active theabrownins were studied. This work can lay a foundation for the application of theabrownins in functional foods, while provide further insights into the use of theabrownins as dietary supplements for lowering lipid levels.

## 2. Materials and Methods 

### 2.1. Materials and Chemicals

*Eurotium cristatum* fermented loose tea, which was prepared in June 2018 [18], was used as a material for theabrownins preparation. Bovine serum albumin (BSA), gallic acid (GA), rutin, simvastatin, dimethyl sulfoxide (DMSO), and oil red O (ORO) were purchased from Sigma-Aldrich Chemical Co. (St. Louis, MO, USA). Egg yolk (S30910, BR grade) was purchased from Yuanye Biotechnology Co. Ltd. (Shanghai, China). Organic reagents of analytical grade including ethanol, chloroform, ethyl acetate, and *n*-butanol were purchased from Chron Chemical Co. Ltd. (Chengdu, China). 

### 2.2. Preparation of Theabrownins

Preparation of theabrownins was conducted according to a previous study [7]. Briefly, *E. cristatum* fermented loose tea was extracted with ultrapure water at 70 °C for 3 h in a water bath, and the solution was then filtered under reduced pressure. The tea infusion was concentrated to a quarter of its original volume at 60 °C using a rotary evaporator, and was then precipitated with three times volume of anhydrous ethanol for 24 h at room temperature. After that, the mixture was centrifuged and the supernatant was used for extracting theabrownins. The supernatant was extracted three times with the equal volume of chloroform, and the aqueous layer was collected and extracted three times with ethyl acetate (1:1, *v*/*v*). Finally, the collected aqueous layer was extracted three times with *n*-butanol (1:1, *v*/*v*), after which it was precipitated with anhydrous ethanol (1:4, *v*/*v*) for 12 h at 4 °C. The precipitate, which is theabrownins (TBs), was collected by centrifugation and then freeze-dried.

### 2.3. Chemical Composition Analysis of Theabrownins

Carbohydrate content of TBs was measured with the phenol-sulfuric acid method using dextrose as a standard [19]. Protein content in TBs was measured by the Bradford’s method using BSA as a standard [20]. Content of total phenols was measured using the Folin–Ciocalteu method, and the values are presented as GA equivalent [21]. Content of total flavonoids was analyzed with a modified spectrophotometric method using rutin as a standard, and the values are represented as rutin equivalent [22].

### 2.4. Preparation of Theabrownins with Different Molecular Weights

TBs were fractionated by ultrafiltration centrifugation through microseps with different molecular weight cutoffs (MWCO) (Amicon Ultra-15, Merck Millipore, Germany). TBs were dissolved in 5 mg/mL in Milli-Q water and then fractionated through microseps with 3k, 10k, and 30k MWCO according to the manufacturer’s instructions. After vacuum freeze drying, four fractions of theabrownins with different molecular weights (Mw): <3 kDa, 3–10 kDa, 10–30 kDa, and >30 kDa, were obtained and were named as TBs-LT3k, TBs-3-10k, TBs-10-30k, and TBs-GT30k, respectively.

### 2.5. UV-Vis Absorption and FT-IR Spectroscopic Analysis of Fractionated Theabrownins

UV-Vis absorption measurements were performed on a U-3900H spectrophotometer (Hitachi, Tokyo, Japan) using a 10-mm quartz cuvette. All theabrownin samples were dissolved in water to 16 µg/mL and their UV-Vis spectra were scanned between 190 and 800 nm using water as a reference.

Flourier transformation infrared (FT-IR) spectra of the samples were recorded on a Nicolet 6700 FT-IR spectrometer (Thermo Fisher Scientific Inc., Waltham, MA, USA) using the KBr-disk technique. Briefly, all theabrownin samples were ground with spectroscopic grade KBr powder and then pressed into 1-mm-thick pellets. The pellets were subjected to FT-IR analysis at a frequency range of 4000 to 400 cm^−1^, a resolution of 4 cm^−1^, and 32 scans per sample.

### 2.6. Hypolipidemic Activity of Fractionated Theabrownins in Zebrafish Model

Wild-type zebrafish (*Danio rerio*) were maintained at 28 °C under a 14 h light:10 h dark cycle. The embryos were produced through natural mating and were maintained at 28 °C in fresh fish water. Larvae at five days after fertilization (5 d.p.f) were fed with 0.1% (m/v) egg yolk as a high-fat diet (HFD) for 48 hours and thereafter were treated with different concentrations of fractionated theabrownins, 0.06 μM simvastatin (positive control), 0.1% DMSO (vehicle control) or fresh fish water, respectively, for another 48 hours. Additionally, larvae zebrafish that were maintained in fresh fish water during the experiment were set as the control group. Zebrafish were placed in the 6-well culture at a density of 10 zebrafish in 4 mL of solution per group of treatment. The lipid levels in these treated zebrafish were measured and compared via ORO staining conducted according to a previous report [23]. Briefly, zebrafish were fixed in 4% paraformaldehyde at 4 °C overnight and were then washed three times with 1 × phosphate-buffered saline (PBS). Subsequently, zebrafish were stained with fresh 3% ORO for 3 h after 30-minute preincubation in 60% isopropanol. Finally, zebrafish were ready for microscopic observation when superfluous ORO was washed off with 60% isopropanol, and six zebrafish were randomly selected for ORO image acquisition in each group. All images were captured by a Leica M205 FA stereomicroscope (Wetzlar, Heidelberg, Germany) operated under the same conditions, and the lipid levels were quantified and normalized to the integral optical density (IOD) values using the Image-pro plus 6.0 software (Media Cybernetics Inc., Rockville, MD, USA). All animal experiments were performed according to the guidelines of the Animal Care and Use Committee of Sichuan University, Chengdu, Sichuan, China, and were approved by the institutional review board of the Medical Faculty at the West China Hospital, Sichuan University, Chengdu, Sichuan, China (Certificate number: SCXK2013-19).

### 2.7. Structural Characterization of TBs-10-30k

#### 2.7.1. Thermal Properties

Thermal properties of TBs-10-30k were analyzed by thermogravimetry (TG) and differential scanning calorimetry (DSC) using TGA/DSC 2 and DSC 1 analyzers (METTLER TOLEDO, Zurich, Switzerland), respectively. In brief, the sample was accurately weighed and placed in the alumina crucible. For TG analysis, the sample was heated at temperatures from 30 to 800 °C under nitrogen gas stream flowed at a rate of 20 mL/min; for DSC analysis, the sample was heated from 30 to 600 °C. The heating rates of both analyses were set at 10 °C/min [24].

#### 2.7.2. X-Ray Diffraction

X-ray diffraction (XRD) of TBs-10-30k was carried out using an Empyrean X-ray diffractometer (PANalytical B.V., Almelo, Netherlands) operated at a voltage and an incident current of 40 kV and 40 mA, respectively. The pattern was recorded at a scanning speed of 1°/min over a diffraction angle (2θ) range of 5 to 65°.

#### 2.7.3. Morphological Characterization

Morphological characteristics of TBs-10-30k were observed under a model JSM-7500F scanning electron microscope (SEM) (JEOL, Tokyo, Japan); prior to the observation, the sample was sputtered with gold. Morphology at nano-level of TBs-10-30k was also observed using a model MultiMode 8 atomic force microscope (AFM; Bruker, Karlsruhe, Germany). Briefly, a small quantity of TBs-10-30k was dissolved in ultrapure water and then diluted to 10 μg/mL. After that, 5 μL of the diluted sample solution was dropped onto a mica surface and then allowed to dry. The obtained AFM images were analyzed by NanoScope Analysis version 1.9 software (Bruker, Karlsruhe, Germany).

#### 2.7.4. Pyrolysis-Gas Chromatography-Mass Spectrometry Analysis

TBs-10-30k sample (0.15 mg) was accurately weighed and then pyrolyzed in a CDS 5000 pyrolyzer (CDS Analytical, Inc., Oxford, Pennsylvania, USA) at 280 °C for 5 s. The pyrolytic products were analyzed by Agilent 5977B GC/MSD instrument (Agilent Technologies Inc., Santa Clara, California, USA) equipped with a DB-5MS column (30 m × 0.25 mm × 0.25 m). The injector temperature was kept at 250 °C, and purified helium was used as the carrier gas flowed at a rate of 1.0 mL/min. The GC oven temperatures were programmed as follows: initial temperature of 40 °C was kept for 1 min; increased to 200 °C at a heating rate of 8 °C/min and maintained for 2 min; and increased to 280 °C at 10 °C/min and held for 5 min. The mass spectrometer was operated in EI ionization mode at 70 eV, and the sample was scanned under the total ion current (TIC) mode from 10 to 800 amu. Temperatures of the quadrupole and ion source were maintained at 150 and 230 °C, respectively. Identities of the pyrolytic products were identified by comparing their mass spectra with the NIST14.L library; their quantities were obtained by peak area normalization and were expressed as the ratio of area of single constituent to total area.

### 2.8. Statistical Analysis

Statistical analysis was performed by SPSS 22.0 software (SPSS Inc., Chicago, IL, USA). Data were expressed as means ± standard deviations. In the zebrafish experiment, significant differences were determined by one-way analysis of variance (ANOVA), followed by the Bonferroni *t*-test post hoc comparisons. Compared to the HFD group, the significant criteria were set to be *p-value < 0.05 and #*p*-value < 0.001, respectively. All the bar plots were generated with GraphPad Prism 7.0 software (GraphPad Software, San Diego, USA).

## 3. Results

### 3.1. Compositions and Molecular Weight Distributions of Theabrownins 

According to the chemical composition analysis, the extracted theabrownins contained 5.62 ± 0.61% of protein, 20.74 ± 0.33% of phenols, 24.62 ± 1.08% of flavonoids, and 16.10 ± 1.07% of carbohydrates. Fractionated by ultrafiltration centrifugation through microseps, theabrownins were separated into four fractions with different molecular weights. The four fractions of theabrownins, TBs-LT3k, TBs-3-10k, TBs-10-30k, and TBs-GT30k, accounted for 49.31 ± 1.30%, 32.46 ± 3.12%, 16.18 ± 2.32%, and 2.05 ± 0.50% in TBs, respectively.

### 3.2. Spectroscopic Characteristics of Theabrownins

**Figure 1:**
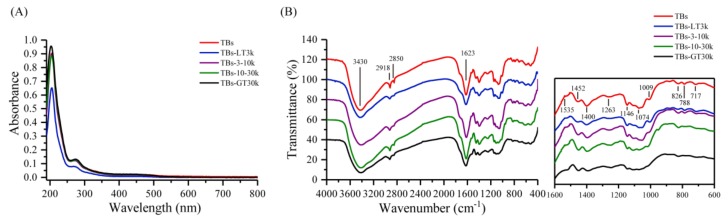
Ultraviolet–visible spectra (**A**) and Flourier transformation infrared spectra (**B**) of theabrownins (TBs) and fractionated theabrownin samples with different molecular weights. TBs-LT3k, TBs-3-10k, TBs-10-30k, and TBs-GT30k represented the theabrownin samples with molecular weights: <3 kDa, 3–10 kDa, 10–30 kDa, and >30 kDa, respectively.

The UV-Vis spectra can be used to identify the compounds containing σ-bonds, π-bonds, lone pair of electrons, chromophores, and aromatic rings. As demonstrated in Figure 1A, all theabrownin samples exhibited similar UV-Vis absorption spectra: one strong absorption band and the other absorption band. The maximum absorption wavelengths were approximately 205 nm and 270 nm. The results indicated the abundance of aromatic C=C content in theabrownins as the π–π^*^ transitions in substituted benzenes or polyphenols occurred in the wavelength region [25]. 

The FT-IR spectra showed that all theabrownin samples exhibited absorption peaks at around 3400 cm^−1^, 2920 cm^−1^, 2850 cm^−1^,1620 cm^−1^, 1535 cm^−1^, 1450 cm^−1^, 1400 cm^−1^,1260–1000 cm^−1^, and 825–710 cm^−1^ (Figure 1B). The strong and broad peak at around 3400 cm^−1^ is the characteristic absorption peak of O–H stretching vibration of polymers [26], indicating that the theabrownins likely are polymerized polyhydroxyl compounds. The absorption peaks at around 2920 cm^−1^ and 2850 cm^−1^ were assigned to the C–H stretching vibration of CH, CH_2_, and CH_3_ groups. The relatively strong absorption peak at around 1620 cm^−1^ is due to the stretching vibration of C=O, which was bonded with aromatic rings. The absorption bands at 1535 cm^−1^, 1450 cm^−1^, 1400 cm^−1^ are the stretching vibration of aromatic C=C [16]. The absorption bands observed at the range of 1260–1000 cm^−1^ are the C–O stretching vibration, and that at around 1260 cm^−1^ can be attributed to the Ar C–OH group [26]. Other absorption bands are due to the presence of C–O–H side group and/or the vibration of C–O–C glycosidic bond [27]. The absorption bands at the range of 825–710 cm^−1^ could be due to the substituents on the benzene rings [16]. These data indicate that the theabrownins exist as polymerized phenolic compounds that are abundant in hydroxy and carboxyl groups and may contain glycosidic bonds.

### 3.3. Hypolipidemic Activity of Fractionated Theabrownins

**Figure 2:**
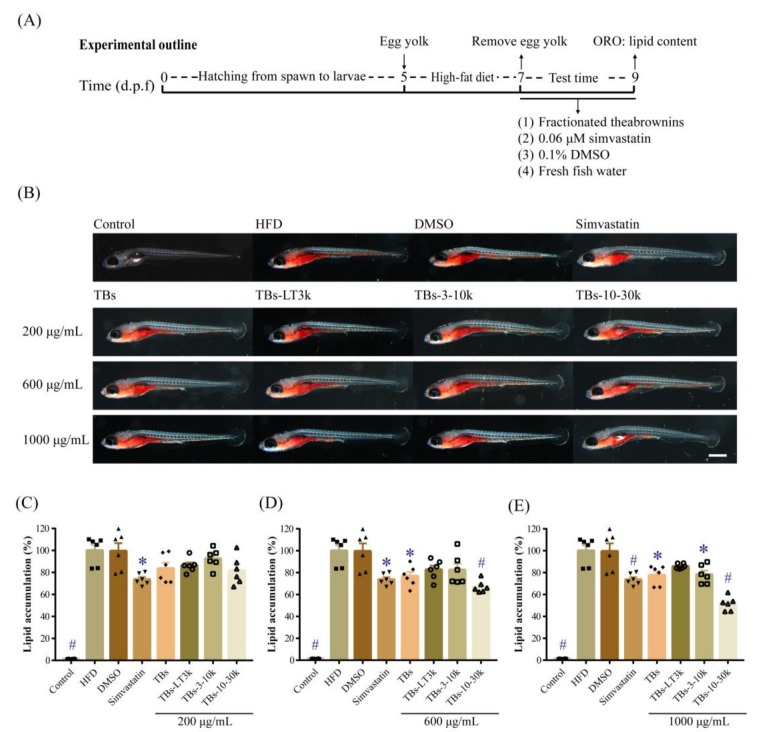
Hypolipidemic effect of theabrownins (TBs) and fractionated theabrownin samples with different molecular weights in high-fat-induced obesity zebrafish. (**A**) Experimental outline of zebrafish model. (**B**) Zebrafish stained with oil red O and visualized under a microscope magnified 30 times. (**C**–**E**) Relative lipid accumulation in zebrafish treated with different concentrations of theabrownins. Data were expressed as mean ± SEM (*n* = 6). *p-value < 0.05 and #*p*-value < 0.001 compared to high-fat diet (HFD) group. HFD, high-fat diet-induced obesity zebrafish. DMSO, vehicle control at 0.1% (*v*/*v*). Simvastatin, positive control at 0.06 μM. TBs-LT3k, TBs-3-10k, and TBs-10-30k represented the fractionated theabrownin samples with molecular weights: <3 kDa, 3–10 kDa, and 10–30 kDa, respectively. Scale bar, 500 μm.

The hypolipidemic effect of the fractionated theabrownins on high-fat induced obese zebrafish was evaluated, and the result is shown in Figure 2. After 48-h egg yolk treatment, lipid levels in gut and blood vessels of high-fat diet induced hyperlipidemia (HFD) zebrafish were significantly increased compared with those of the control group untreated with egg yolk. The lipid level of HFD group was set as 100%, and lipid accumulations in all other groups were expressed as relative lipid levels to that of HFD group. The results showed that the lipid levels of zebrafish treated with different concentrations of the fractionated theabrownins dropped to different extents. As shown in Figure 2C, the lipid levels of hyperlipidemia zebrafish treated with TBs, TBs-LT3k, TBs-3-10k, and TBs-10-30k at 200 μg/mL were reduced to 83.61%, 86.37%, 92.73%, and 81.73%, respectively (*p* > 0.05). Lipid levels of zebrafish treated with TBs-LT3k and TBs-3-10k (600 μg/mL) were decreased to 82.74% and 82.56%, respectively (*p* > 0.05), and those of zebrafish treated with TBs and TBs-10-30k were significantly decreased to 76.80% (*p* < 0.05) and 67.12% (*p* < 0.001), respectively (Figure 2D). We further observed that treatment with fractionated theabrownins at 1000 μg/mL was only slightly more effective in lowering lipid levels: treatments with TBs, TBs-LT3k, TBs-3-10k, and TBs-10-30k caused the lipid levels of zebrafish to decrease to only 77.79%, 85.72%, 78.43%, and 51.57%, respectively (Figure 2E). Simvastatin, an HMG-CoA reductase inhibitor commonly used as a hypolipidemic drug in clinical treatment, significantly reduced the lipid level of hyperlipidemia zebrafish to 73.79%; conversely, the vehicle DMSO at a final concentration of 0.1% (*v*/*v*) had no effect on the lipid level of zebrafish. Comparing the hypolipidemic effects of all fractionated theabrownins showed that TBs-10-30k was most effective in lowering lipid content of hyperlipidemia zebrafish. Hence, the structure characteristics of TBs-10-30k were further analyzed in subsequent experiment.

### 3.4. Structure Characteristics of TBs-10-30k

#### 3.4.1. Thermal Properties

**Figure 3:**
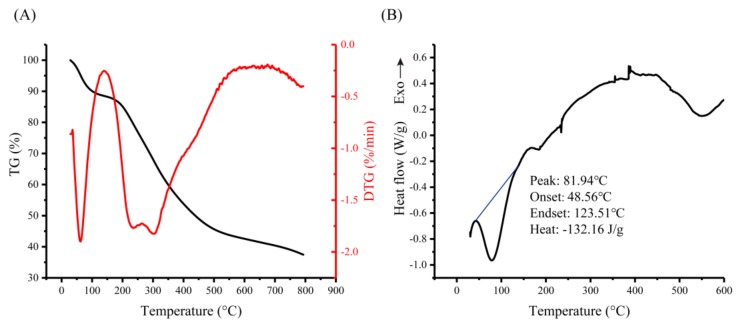
Thermogravimetry/Differential thermogravimetry analysis (**A**) and differential scanning calorimetry (**B**) analysis results of TBs-10-30k.

The thermal behavior of TBs-10-30k was analyzed by thermogravimetric curve, and the thermal degradation and its first derivates are depicted in Figure 3A. TBs-10-30k exhibited two-stage weight loss. Initial weight loss occurred at the temperature range of 30 to 138 °C, which is the loss of free and bound water [24,28]. Weight loss (39.45%) at the temperature range of 200 to 500 °C can be ascribed to TBs-10-30k decomposition. According to the weight loss derivative curve (DTG), three maximum weightlessness rates occurred at 62 °C, 235 °C, and 305 °C, respectively. The DSC curve illustrated in Figure 3B shows an obvious endothermic peak at 81.94 °C with an energy requirement of 132.16 J/g, which may be caused by the dehydration or loss of peripheral chains of TBs-10-30k and dehydroxylation reactions [24,28]. Generally, the denaturation temperature peak and enthalpy of transition can reflect sample’s ability to retain water, which is related to the sample hydrophilic groups [29]. Comparing the results with those reported previously, it can be concluded that TBs-10-30k is suitable for applications in thermal processing conditions [30].

#### 3.4.2. X-Ray Diffraction

**Figure 4:**
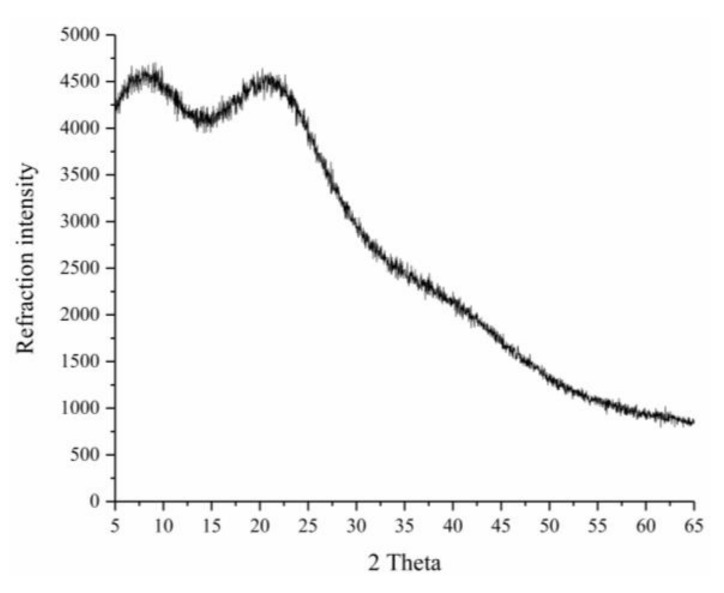
X-ray diffraction pattern of TBs-10-30k.

XRD is a powerful analytical tool widely used to determine the crystallinity of samples, such as polysaccharides and starch. Crystalline materials generally exhibit sharp narrow diffraction peaks, whereas amorphous materials exhibit broad diffraction peaks [31]. The XRD pattern of TBs-10-30k recorded at 2θ angles of 5° to 65° is shown in Figure 4. No obvious sharp narrow diffraction peaks were observed, and the XRD pattern had a broad “bun-like” shape. This result indicates that TBs-10-30k is an amorphous polymer. 

#### 3.4.3. Morphological Characteristics

**Figure 5:**
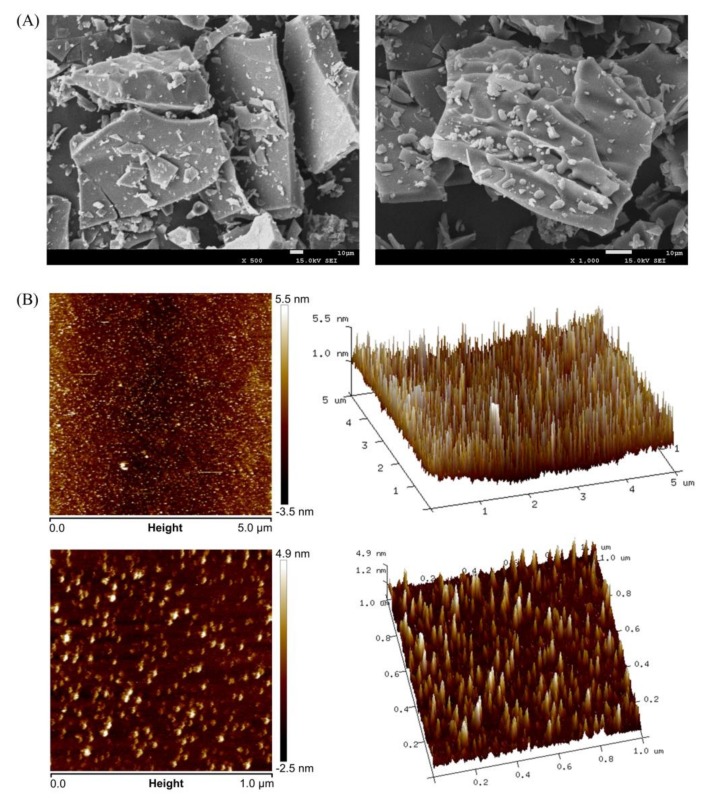
Morphological characteristics of TBs-10-30k. (**A**) Images magnified by scanning electron microscopy at 500 and 1000 times, respectively. (**B**) Planar images and corresponding three-dimensional images of TBs-10-30k obtained by atomic force microscopy. The scanning range is 5 × 5 μm and 1 × 1 μm, respectively.

Scanning electron microscopy is a qualitative tool that is widely used to analyze the surface morphology of various samples. The SEM images depicted in Figure 5A showed that TBs-10-30k was in aggregated state or amorphous polymer, which is consistent with that of XRD; it also mainly had slice shape and relatively smooth surface. 

Atomic force microscopy is a powerful and visual tool that can be used to analyze the morphologies of samples under natural conditions. The three-dimensional structure and microscopic characteristics of TBs-10-30k observed by AFM are depicted in Figure 5B. We observed that TBs-10-30k had island-like structure, because of the aggregation of theabrownin molecules formed by intermolecular and intramolecular hydrogen bonds between hydroxyl groups on the theabrownin chains and water molecules [31]. This result is consistent with that from FT-IR, which showed that theabrownins exhibited strong characteristic absorption band caused by O–H stretching vibration. Rq and Ra are roughness parameters obtained from NanoScope Analysis software; they represent the root mean square average of height deviations taken from the mean image data plane and the arithmetic average of the absolute values of the surface height deviations measured from the mean plane, respectively. Within the scanning area of 5 × 5 μm, Rq and Ra values of TBs-10-30k were 1.26 nm and 0.966 nm, respectively.

#### 3.4.4. Pyrolysis-Gas Chromatography-Mass Spectrometry Analysis

Achieving fine structures of theabrownins is difficult because of their complex compositions. In the present study, pyrolysis-gas chromatography-mass spectrometry (Py-GC-MS) analysis was applied to study the pyrolytic products of TBs-10-30k. As summarized in Table 1, TBs-10-30k pyrolyzed at 280 °C yielded 39 compounds. Main pyrolytic products with contents of higher than 1.0% included hexadecanoic acid (33.72%), phenol (14.90%), eicosane (12.95%), octadecane (6.04%), tetracosane (3.72%), octadecanoic acid (3.52%), nonanal (2.22%), 1,3-diisocyanato-2-methylbenzene (1.82%), 1-chlorododecane (1.76%), tetradecanoic acid (1.73%), decanal (1.31%), 2-pyranone-6-carboxylic acid (1.21%), pentadecanoic acid (1.19%), 2,4-bis(1,1-dimethylethyl)-phenol (1.16%), and diisooctyl phthalate (1.12%). Some fatty acids, such as hexadecanoic acid (1.42-4.03%) and octadecanoic acid (0.58%) detected in the pyrolytic products of TBs-10-30k, also existed in the pyrolytic products of theabrownins extracted from Pu-erh tea [16]. In addition, 4-heptyltridecyl-fumaric acid (4.24%) and (E)-9-octadecenoic acid (2.78%), found in the pyrolytic products of TBs-10-30k, are parts of the pyrolytic products of theabrownins from Zijuan tea [17]. These fatty acids are suggested to be produced from the lipids and their oxidative products during the pyrolysis process, and the types of teas from which theabrownins were prepared and pyrolytic temperatures can lead to different pyrolytic products [16,17]. High contents of pyrolytic phenolic compounds detected in TBs-10-30k (as has been observed in previous reports) indicate that theabrownins are partly polymerized and partly oxidized from catechins, theaflavins, and thearubigins. Some compounds, such as l-gala-l-ido-octose, melibiose, and 2,3-dihydrobenzofuran, were detected, which were suggested to be pyrolyzed from saccharides. The pyrolytic products of TBs-10-30k are similar to those previously reported; the products mainly contained phenols, acids, organic hydrocarbons, aromatic compounds, ketones, alcohols, aldehydes, and nitrogen-compounds [16,17]. However, caffeine, which has been reported to be abundant in pyrolytic products of theabrownins extracted from Pu-erh tea [16], was not detected in TBs-10-30k. This may be due to that the tea samples used in this study were different from those used in the previous reports. Overall, we conclude that TBs-10-30k (theabrownins) is mainly composed of phenols, lipids, saccharides, and proteins.

## 4. Discussion

It is known that obesity and hyperlipidemia are less likely found in the ethnic groups in southwestern China where dark tea has been largely consumed for a long period of time [32]. Theabrownins have been shown to be the main functional compounds involving lipid-lowering bioactivity of post-fermented dark tea. Accordingly, production of theabrownins by microbial fermentation or fungal enzymes [33,34], and development of high-theabrownins tea products have received considerable attention [6,35]. Nevertheless, isolation and purification of theabrownins can be technically challenging, and in-depth researches on physicochemical property and bioactivity of theabrownins are rarely conducted. In the present study, hypolipidemic and structural characteristics of theabrownins isolated from *E. cristatum* fermented loose tea were studied. One of the theabrownin fractions, TBs-10-30k, which had high molecular weight, was observed to be most effective in lowering lipid levels compared to other fractions. In a report by Liu et al. in which NMR-based metabolomic technique was used, Pu-erh tea theabrownins with a molecular weight of greater than 50 kDa were found to accelerate lipid catabolism in rats. They speculated that this might be due to elevated enzyme activities of carnitine palmitoyltransferases I and II, the crucial enzymes in the catabolism of fatty acids [12]. By contrast, other reports have shown that lipid-lowering effect of theabrownins from Pu-erh tea is associated with enhanced activity of hepatic lipase and hormone-sensitive triglyceride lipase (HSL), as well as increased expression of HSL mRNA in liver and epididymis tissues of rats [13]. Moreover, theabrownins promotes the conversion of cholesterol to bile acids in hepatocytes and stimulates fecal excretion of cholesterol and its metabolites [14]. The reduction of lipid levels in zebrafish caused by theabrownins TBs-10-30k may be through similar mechanisms; however, detailed mechanisms should be further investigated because molecular weight and origin of TBs-10-30k were different from those previously reported. In addition, one report has shown that pure theabrownin, a highly oxidized product from tea polyphenols, can alleviate accumulation of lipids in hepatic cells and reduce weight of white adipose tissue of rats through regulating expression levels of genes involving in fatty acid synthesis, fatty acid oxidation, diet-derived cholesterol transportation, and bile acids formation [4]. These reports show that despite of their different origins and molecular weights, theabrownins exhibit hypolipidemic bioactivity through different metabolic pathways. The detailed hypolipidemic mechanisms of TBs-10-30k should, however, be further investigated. 

Unravelling the structure of theabrownins is necessary for the understanding of their health-promoting effects. During fermentation of different tea products, catechins can be oxidized and transformed into novel dimeric (theaflavins), oligomeric (thearubigins), and polymeric compounds. The structures of theaflavins contain a benzotropolone skeleton, which is the core pharmacophore and is formed by co-oxidation of selected pairs of catechins: one with a *vic*-trihydroxyphenyl moiety (pyrogallol) and the other with an *ortho*-dihydroxyphenyl structure (catechol) [36,37]. Owing to the complexity of the multi-molecular reaction system and multiple reaction sites of catechins and its derivates, the structural characterization of thearubigins have been done poorly. Based on a few reports, thearubigins are heterogeneous polymers of catechins, of which the 3-OH group is likely esterified by gallic acid, or derivates of catechins dimers in which the gallate part has been condensed with “B-ring” catechins [5,36,37]. As post-fermented dark tea undergoes a more complex microbial fermentation process, structures of theabrownins become much more difficult to analyze because they may be oxidized, polymerized, condensed, and coupled from catechins, theaflavins, thearubigins, and their derivates with other compounds that exist in the fermentation process. Holistically, these results are in line with our speculation, in which theabrownins are amorphous and heterogeneous polymerized phenolic compounds containing an abundance of hydroxy and carboxyl groups, as well as lipids, saccharides, and proteins. Elucidating theabrownin structures should facilitate the researches on benefits of theabrownins or those on consumption of dark tea and dark tea-based products. Theabrownins structures can also provide further insights into how they lower lipid levels, which may have implication in dietary supplements.

## 5. Conclusions

In the present study, the structural characteristics and hypolipidemic activity of theabrownins isolated from *Eurotium cristatum*-fermented loose tea were investigated for the first time. The UV-Vis and IR spectra showed that all theabrownin samples exhibited similar characteristics, demonstrating that they were polymerized phenolic substances with abundant hydroxy and carboxyl groups. In addition, all theabrownin samples showed hypolipidemic activity in high-fat-induced obese zebrafish, and TBs-10-30k showed the highest hypolipidemic activity. At 1000 μg/mL, TBs-10-30k was able to decrease the lipid level of high-fat zebrafish to 51.57%. The structural analysis revealed that TBs-10-30k was an amorphous and heterogeneous polymer mainly composed of phenols, lipids, saccharides, and proteins. Moreover, TG and DSC analyses showed that TBs-10-30k had good thermostability. This work can facilitate future identification of theabrownins and might provide insights into the applications of theabrownins in functional foods.

## Figures and Tables

**Table 1:** GC-MS analysis of TBs-10-30k pyrolytic products at 280 °C.

No.	Rt (min) ^1^	Compound	CAS	Content (%) ^2^	SI ^3^
1	7.67	Phenol	108-95-2	14.90	25
2	8.47	4-Amino-6-hydroxypyrimidine	1193-22-2	0.91	60
3	9.14	2-Acetyl pyrrole	1072-83-9	0.38	58
4	9.54	3-Methylhex-3-en-2-one	1187-80-0	0.36	38
5	9.94	Nonanal	124-19-6	2.22	91
6	10.15	3,4-Dimethyl-3-pyrrolin-2-one	4030-22-2	0.59	64
7	11.17	2-Pyranone-6-carboxylic acid	672-67-3	1.21	58
8	11.60	9-[1-Hydroxy-2-dibutylaminoethyl]-10-chloro phenanthrene	52979-76-7	0.72	78
9	11.83	Decanal	112-31-2	1.31	91
10	12.05	2,3-Dihydrobenzofuran	496-16-2	0.24	76
11	12.86	Nonanoic acid	112-05-0	0.42	53
12	13.47	Indole	120-72-9	0.22	46
13	14.42	1,3-Diisocyanato-2-methylbenzene	91-08-7	1.82	98
14	15.20	7-Amino-1,3-dihydro-indol-2-one	1000303-02-6	0.61	64
15	15.30	1,3-Dihydro-5-methyl-2H-benzimidazol-2-one	5400-75-9	0.67	91
16	16.27	1-Chlorododecane	112-52-7	1.76	91
17	16.50	Dihydrocoumarin, 4,4,5,7,8-pentamethyl	39170-97-3	0.53	50
18	16.93	2,4-Bis(1,1-dimethylethyl)-phenol	96-76-4	1.16	96
19	17.58	Dodecanoic acid	143-07-7	0.35	64
20	18.24	Pentanoic acid, 2,2,4-trimethyl-3-carboxyisopropyl, isobutyl ester	1000140-77-5	0.97	91
21	19.30	Oxalic acid, cyclobutyl octadecyl ester	1000309-70-8	0.51	38
22	19.90	l-Gala-l-ido-octose	1000130-12-1	0.56	32
23	20.40	Tetradecanoic acid	544-63-8	1.73	99
24	20.87	N-Methylmaleimide	930-88-1	0.44	38
25	20.94	Octadecane	593-45-3	6.04	98
26	21.13	1-Hexadecanol, 2-methyl-	2490-48-4	0.27	18
27	21.34	Melibiose	585-99-9	0.31	32
28	21.75	Pentadecanoic acid	1002-84-2	1.19	98
29	22.00	Phthalic acid, butyl 2-methylpent-3-yl ester	1000356-90-8	0.40	98
30	22.45	Quinoline-5,8-dione-6-ol, 7-[[(4-cyclohexylbutyl) amino] methyl]-	1000252-66-9	0.30	37
31	23.05	Palmitoleic acid	373-49-9	0.74	43
32	23.48	Hexadecanoic acid	57-10-3	33.72	99
33	25.97	Butyl 9-tetradecenoate	1000336-51-4	0.41	53
34	26.27	Octadecanoic acid	57-11-4	3.52	99
35	27.82	Tetracosane	646-31-1	3.72	98
36	29.73	Eicosane	112-95-8	12.95	97
37	30.21	Diisooctyl phthalate	131-20-4	1.12	76
38	32.27	2-Ethylacridine	55751-83-2	0.34	30
39	34.52	Benzo[h]quinoline, 2,4-dimethyl-	605-67-4	0.35	46

^1^ Rt, retention time of pyrolytic product. ^2^ The relative contents of pyrolytic products were calculated by peak area normalization, and results are expressed as the ratio of single constituent area to total area. ^3^ SI, index of similarity.

## References

[1] Hasumura T., Shimada Y., Kuroyanagi J., Nishimura Y., Meguro S., Takema Y., Tanaka T. (2012). Green tea extract suppresses adiposity and affects the expression of lipid metabolism genes in diet-induced obese zebrafish. Nutr. Metab..

[2] Huang H.C., Lin J.K. (2012). Pu-erh tea, green tea, and black tea suppresses hyperlipidemia, hyperleptinemia and fatty acid synthase through activating AMPK in rats fed a high-fructose diet. Food Funct..

[3] Ikeda I., Yamahira T., Kato M., Ishikawa A. (2010). Black-Tea Polyphenols Decrease Micellar Solubility of Cholesterol in Vitro and Intestinal Absorption of Cholesterol in Rats. J. Agric. Food Chem..

[4] Wang S., Huang Y., Xu H., Zhu Q., Lu H., Zhang M., Hao S., Fang C., Zhang D., Wu X. (2017). Oxidized tea polyphenols prevent lipid accumulation in liver and visceral white adipose tissue in rats. Eur. J. Nutr..

[5] Kuhnert N. (2010). Unraveling the structure of the black tea thearubigins. Arch. Biochem. Biophys..

[6] Wang Q., Belščak-Cvitanović A., Durgo K., Chisti Y., Gong J., Sirisansaneeyakul S., Komes D. (2018). Physicochemical properties and biological activities of a high-theabrownins instant Pu-erh tea produced using *Aspergillus tubingensis*. LWT Food Sci. Technol..

[7] Chen D., Sun J., Dong W., Shen Y., Xu Z. (2018). Effects of polysaccharides and polyphenolics fractions of Zijuan tea (*Camellia sinensis var. kitamura*) on α-glucosidase activity and blood glucose level and glucose tolerance of hyperglycaemic mice. Int. J. Food Sci. Technol..

[8] Liu T., Xiang Z., Chen F., Yin D., Huang Y., Xu J., Hu L., Xu H., Wang X., Sheng J. (2018). Theabrownin suppresses in vitro osteoclastogenesis and prevents bone loss in ovariectomized rats. Biomed. Pharmacother..

[9] Jin W., Zhou L., Yan B., Yan L., Liu F., Tong P., Yu W., Dong X., Xie L., Zhang J. (2018). Theabrownin triggers DNA damage to suppress human osteosarcoma U2OS cells by activating p53 signalling pathway. J. Cell. Mol. Med..

[10] Zhou L., Wu F., Jin W., Yan B., Chen X., He Y., Yang W., Du W., Zhang Q., Guo Y. (2017). Theabrownin Inhibits Cell Cycle Progression and Tumor Growth of Lung Carcinoma through c-myc-Related Mechanism. Front. Pharmacol..

[11] Wu F., Zhou L., Jin W., Yang W., Wang Y., Yan B., Du W., Zhang Q., Zhang L., Guo Y. (2016). Anti-Proliferative and Apoptosis-Inducing Effect of Theabrownin against Non-small Cell Lung Adenocarcinoma A549 Cells. Front. Pharmacol..

[12] Liu J., Peng C.-X., Gao B., Gong J.-S. (2016). Serum metabolomics analysis of rat after intragastric infusion of Pu-erh theabrownin. J. Sci. Food Agric..

[13] Gong J., Peng C., Chen T., Gao B., Zhou H. (2010). Effects of Theabrownin from Pu-erh Tea on the Metabolism of Serum Lipids in Rats: Mechanism of Action. J. Food Sci..

[14] Peng C.-X., Wang Q.-P., Liu H.-R., Gao B., Sheng J., Gong J. (2013). Effects of Zijuan pu-erh tea theabrownin on metabolites in hyperlipidemic rat feces by Py-GC/MS. J. Anal. Appl. Pyrolysis.

[15] Wang Q.-P., Peng C.-X., Gao B., Gong J.-S. (2012). Influence of large molecular polymeric pigments isolated from fermented Zijuan tea on the activity of key enzymes involved in lipid metabolism in rat. Exp. Gerontol..

[16] Peng C.-X., Liu J., Liu H.-R., Zhou H.-J., Gong J.-S. (2013). Influence of different fermentation raw materials on pyrolyzates of Pu-erh tea theabrownin by Curie-point pyrolysis-gas chromatography–mass spectroscopy. Int. J. Biol. Macromol..

[17] Gong J., Zhang Q., Peng C., Fan J., Dong W. (2012). Curie-point pyrolysis–gas chromatography–mass spectroscopic analysis of theabrownins from fermented Zijuan tea. J. Anal. Appl. Pyrolysis.

[18] Xiao Y., Zhong K., Bai J.-R., Wu Y.-P., Zhang J.-Q., Gao H. (2020). The biochemical characteristics of a novel fermented loose tea by *Eurotium cristatum* (MF800948) and its hypolipidemic activity in a zebrafish model. LWT Food Sci. Technol..

[19] Albalasmeh A.A., Berhe A.A., Ghezzehei T.A. (2013). A new method for rapid determination of carbohydrate and total carbon concentrations using UV spectrophotometry. Carbohydr. Polym..

[20] Bradford M.M. (1976). A rapid and sensitive method for the quantitation of microgram quantities of protein utilizing the principle of protein-dye binding. Anal. Biochem..

[21] Velioglu Y.S., Mazza G., Gao L., Oomah B.D. (1998). Antioxidant Activity and Total Phenolics in Selected Fruits, Vegetables, and Grain Products. J. Agric. Food Chem..

[22] Ordoñez A.A.L., Gomez J.D., Vattuone M.A., Isla M.I. (2006). Antioxidant activities of *Sechium edule* (Jacq.) Swartz extracts. Food Chem..

[23] Chen K., Wang C.Q., Fan Y.Q., Xie Y.S., Yin Z.F., Xu Z.J., Zhang H.L., Cao J.T., Han Z.H., Wang Y. (2015). Optimizing methods for the study of intravascular lipid metabolism in zebrafish. Mol. Med. Rep..

[24] Guo M.Z., Meng M., Duan S.Q., Feng C.C., Wang C.L. (2019). Structure characterization, physicochemical property and immunomodulatory activity on RAW264.7 cells of a novel triple-helix polysaccharide from Craterellus cornucopioides. Int. J. Biol. Macromol..

[25] Chen J., Gu B., LeBoeuf E.J., Pan H., Dai S. (2002). Spectroscopic characterization of the structural and functional properties of natural organic matter fractions. Chemosphere.

[26] Pretsch E., Buhlmann P., Badertscher M. (2009). Structure Determination of Organic Compounds: Tables of Spectral Data.

[27] Yang X., Huang M., Qin C., Lv B., Mao Q., Liu Z.J. (2017). Structural characterization and evaluation of the antioxidant activities of polysaccharides extracted from Qingzhuan brick tea. Int. J. Biol. Macromol..

[28] Nawrocka A., Szymańska-Chargot M., Miś A., Wilczewska A.Z., Markiewicz K.H. (2017). Effect of dietary fibre polysaccharides on structure and thermal properties of gluten proteins—A study on gluten dough with application of FT-Raman spectroscopy, TGA and DSC. Food Hydrocoll..

[29] Trigui I., Yaich H., Sila A., Cheikh-Rouhou S., Bougatef A., Blecker C., Attia H., Ayadi M.A. (2018). Physicochemical properties of water-soluble polysaccharides from black cumin seeds. Int. J. Biol. Macromol..

[30] Wang L., Zhang B., Xiao J., Huang Q., Li C., Fu X. (2018). Physicochemical, functional, and biological properties of water-soluble polysaccharides from *Rosa roxburghii* Tratt fruit. Food Chem..

[31] Ji X., Liu F., Peng Q., Wang M. (2018). Purification, structural characterization, and hypolipidemic effects of a neutral polysaccharide from *Ziziphus Jujuba cv. Muzao*. Food Chem..

[32] Li Q., Liu Z., Huang J., Luo G., Liang Q., Wang D., Ye X., Wu C., Wang L., Hu J. (2013). Anti-obesity and hypolipidemic effects of Fuzhuan brick tea water extract in high-fat diet-induced obese rats. J. Sci. Food Agric..

[33] Wang Q., Gong J., Chisti Y., Sirisansaneeyakul S. (2016). Production of theabrownins using a crude fungal enzyme concentrate. J. Biotechnol..

[34] Wang Q., Gong J., Chisti Y., Sirisansaneeyakul S. (2015). Fungal Isolates from a Pu-Erh Type Tea Fermentation and Their Ability to Convert Tea Polyphenols to Theabrownins. J. Food Sci..

[35] Wang Y., Zhang M., Zhang Z., Lu H., Gao X., Yue P. (2017). High-theabrownins instant dark tea product by Aspergillus niger via submerged fermentation: α-glucosidase and pancreatic lipase inhibition and antioxidant activity. J. Sci. Food Agric..

[36] Li S.M., Lo C.Y., Pan M.H., Lai C.S., Ho C.T. (2013). Black tea: Chemical analysis and stability. Food Funct..

[37] Menet M.-C., Sang S., Yang C.S., Ho C.-T., Rosen R.T. (2004). Analysis of Theaflavins and Thearubigins from Black Tea Extract by MALDI-TOF Mass Spectrometry. J. Agric. Food Chem..

